# Transcriptome-Wide Identification of WRKY Transcription Factors and Their Expression Profiles under Different Types of Biological and Abiotic Stress in *Pinus massoniana* Lamb

**DOI:** 10.3390/genes11111386

**Published:** 2020-11-23

**Authors:** Sheng Yao, Fan Wu, Qingqing Hao, Kongshu Ji

**Affiliations:** Key Laboratory of Forestry Genetics & Biotechnology of Ministry of Education, Co-Innovation Center for Sustainable Forestry in Southern China, Nanjing Forestry University, Nanjing 210037, China; yaosheng0817@163.com (S.Y.); eiknarf@126.com (F.W.); hjh19960812@126.com (Q.H.)

**Keywords:** *P. massoniana*, WRKY, biological and abiotic stress, expression pattern

## Abstract

*Pinus massoniana* Lamb, an economically important conifer tree, is widely distributed in China. WRKY transcription factors (TFs) play important roles in plant growth and development, biological and abiotic stress. Nevertheless, there is little information about the WRKY genes in *P. massoniana*. By searching for conserved WRKY motifs in transcriptomic RNA sequencing data for *P. massoniana*, 31 sequences were identified as WRKY TFs. Then, phylogenetic and conserved motif analyses of the WRKY family in *P. massoniana*, *Pinus taeda* and *Arabidopsis thaliana* were used to classify WRKY genes. The expression patterns of six *PmWRKY* genes from different groups were determined using real-time quantitative PCR for 2-year-old *P. massoniana* seedings grown in their natural environment and challenged by phytohormones (salicylic acid, methyl jasmonate, or ethephon), abiotic stress (H_2_O_2_) and mechanical damage stress. As a result, the 31 *PmWRKY* genes identified were divided into three major groups and several subgroups based on structural and phylogenetic features. *PmWRKY* genes are regulated in response to abiotic stress and phytohormone treatment and may participate in signaling to improve plant stress resistance. Some *PmWRKY* genes behaved as predicted based on their homology with *A. thaliana* WRKY genes, but others showed divergent behavior. This systematic analysis lays the foundation for further identification of WRKY gene functions to aid further exploration of the functions and regulatory mechanisms of *PmWRKY* genes in biological and abiotic stress in *P. massoniana*.

## 1. Introduction

Plants are capable of managing various types of stress during their life cycle and have developed several mechanisms to adapt to biotic and abiotic stress. Families of transcription factors (TFs) play an important role in stress response by regulating the expression of target genes, interacting with specific cis-acting elements present in their promoter regions, and acting as activators or repressors in plant stress responses [1,2]. WRKY transcription factors (TFs) constitute one of the largest families of transcription regulators in plants. The most prominent feature of the WRKY protein is its highly conserved WRKY domain, which is composed of 60 amino acids and has a "WRKYGQK" amino acid motif at the N-terminus [3,4]. In a few WRKY proteins, the conserved “WRKYGQK” amino acid sequence can also be replaced by various other forms, such as “WRKYDHK”, “WRKYDQK” and “WRKYGKK” [5]. In addition, the WRKY protein contains a C2H2- or C2HC-type zinc finger structure [5]. Based on structural differences in the WRKY domain and zinc finger, WRKY TFs have been divided into three main groups [5]. Members of the first group contain two WRKY domains and one C2H2 zinc finger motif, those of the second group include a WRKY domain and a C2H2 zinc finger motif, and those of the third group have a C2HC zinc finger motif [6].

Since the initial report of the first WRKY protein in sweet potato, WRKYs have been widely identified and analysed throughout the plant lineage [7]. Previous studies have indicated that some WRKY TFs also play key roles in increasing or decreasing plant resistance to biological and abiotic stresses, and these TFs were found to differ from one another with respect to their inducibilities and functions [6]. For example, the *StWRKY45* gene showed high expression under low-phosphorus stress in *Stylosanthes guianensis*, so it was speculated that the *StWRKY45* gene might be involved in the response to low-phosphorus stress [8]. In transgenic *Arabidopsis thaliana*, *AtWRKY15* and *AtWRKY17* enhance tolerance to salt and osmotic stress [9]. *AtWRKY6* was significantly induced by mechanical damage in *A. thaliana* [10]. Studies have shown that the WRKY protein affects the signal transduction process mediated by plant hormones, such as abscisic acid (ABA) and salicylic acid (SA) [10,11]. For instance, *EgrWRKY70*, *EgrWRKY46* and *EgrWRKY53* have synergistic effects with the hormones SA and methyl jasmonate (MeJA) and play a positive regulatory role in *Eucalyptus grandis* [12].

*Pinus massoniana* is a tree species of Pinaceae that is widely distributed and is an important raw material in industrial and agricultural production. *P. massoniana* is highly resistant to drought and barren land and exhibits high levels of secondary metabolic changes [13]. The biosynthetic regulators of secondary metabolism play a very important role in plant adaptation to environmental stress. *P. massoniana* is, therefore, a good specimen for exploring stress response mechanisms in woody plants. Hence, the identification of functional genes in *P. massoniana* is of great interest. In addition, as *Pinus massoniana* is widely distributed in a variety of environments, facing different stresses and lacking of management, the economic losses caused by biological and abiotic stresses are very serious. WRKY genes is one of the largest family of transcription factors in plants, and play a key role in the regulation of plant biotic and abiotic stress response. Therefore, functional identification of the WRKY family in *P. massoniana* is necessary. Some chromosomal WRKY homologues have evolved to perform different functions in different species, so it is difficult to draw conclusions by comparing WRKY genes and homologous chromosomes [6]. The increasing number of sequenced genomes has facilitated genome-wide identification and analysis of WRKY genes on a large scale in some species, aiding the understanding of their biological functions [14]. The genome of *P. massoniana* is more than 200 times larger than that of *A. thaliana*, so it is difficult to rapidly obtain the sequence; therefore, we used the transcriptome to identify the WRKY gene family here. The WRKY gene family may be crucial for the remarkable tolerance of *P. massoniana*, and we identified 31 *PmWRKY* genes based on RNA-seq, and the classification, domain structures, subcellular localization prediction, and expression patterns of *PmWRKY*s were analysed using bioinformatic methods. In this study, we analysed the transcript levels and expression patterns of six selected *PmWRKY* genes under the effects of stress and phytohormone treatments. This research will not only enrich the understanding of the molecular regulation mechanism of the WRKY gene family but also provide useful information for further exploring the function and regulatory mechanism of *PmWRKY* genes under biological and abiotic stress in *P. massoniana*.

## 2. Materials and Methods 

### 2.1. Selection of PmWRKY Genes

Transcriptome data for Masson pine were derived from the previously determined CO_2_ stress transcriptome [15] and young shoots transcriptome (PRJNA655997). Based on the WRKY domain (PF03106), the hidden Markov model file was downloaded from the Pfam protein family database. HMMER 3.0 was selected to search for WRKY genes in the databases of Iso-Seq. The default parameters were used for screening, and the E value was set to E < 10^−3^ [14]. Pfam (http://pfam.xfam.org/) and CD-search (https://www.ncbi.nlm.nih.gov/cdd/) were used to screen out protein sequences of Masson pine with the WRKY domain. Finally, sequences with complete WRKY domains were selected and sequences more than 97% similarity between different databases were deleted. 

### 2.2. Sequence Analysis

The molecular weights and isoelectric points of the identified *PmWRKY* proteins were obtained using tools from the ExPASy website. The subcellular localization of *PmWRKY* proteins was predicted and analyzed with CELLO (http://cello.life.nctu.edu.tw/) and PSORT (https://psort.hgc.jp/). In addition, we also located the NLS of sequences with cNLS Mapper (http://nls-mapper.iab.keio.ac.jp/) and NLStradamus (http://www.moseslab.csb.utoronto.ca). Based on the *AtWRKY* classification in *A. thaliana*, 65 *AtWRKY* proteins from groups I, II, or III were selected and downloaded from the *Arabidopsis* Information Resource (TAIR) to analyze the phylogenetic relationships among the *AtWRKY* proteins, the 21 *PtWRKY* proteins and the 31 *PmWRKY* proteins in this study [6,16]. The deduced amino acid sequences of *PmWRKY* domains spanning ~60 amino acids were aligned with *AtWRKY*s and *PtWRKY*s using the Clustal program (version 2.1). A phylogenetic tree of the deduced amino acid sequences from WRKY domains in *A. thaliana*, *P. taeda* and *P. massoniana* was constructed using the maximum likelihood method, and a graphical representation was produced with the help of MEGA-X software using 1000 bootstrap replicates [4,17]. The multiple sequence alignment (MSA) of the 60-amino-acid conserved region of *P. massoniana* was visualized using DNAMAN. The MSA included conserved regions of WRKY members representing each group and subgroup from *A. thaliana* and *P. taeda* as references [18,19]. The conserved motifs of *PmWRKY* proteins were analyzed using Multiple Expectation Maximization for Motif Elicitation (MEME: http://meme-suite.org/tools/meme) with the following parameters: minimum and maximum motif widths, 6 and 50, respectively; and maximum number of motifs, 10 [4,20].

### 2.3. Subcellular Localization

*PmWRKY6* and *PmWRKY7* were selected for a transient expression experiment. The coding DNA sequence (CDS) regions were inserted into a pJIT166-GFP expression vector. Transient expression vectors (35S::*PmWRKY6*-GFP and 35S::*PmWRKY7*-GFP) containing green fluorescent protein (GFP) were transferred into the leaves of *Nicotiana benthamiana* following the method of Li [21]. The fluorescence signals were observed with an LSM 710 confocal microscope (Zeiss, Jena, Germany).

### 2.4. Plant Material and Treatments

Two-year-old Masson pine seedlings obtained from the seed orchard of the Baisha state-owned forest farm, Shanghang, Fujian Province, China, were used in this study. To study the expression level of PmWRKY, we subjected the Masson pine seedlings to five treatments. The five treatments included abiotic stress and hormone treatments, namely, mechanical injury, 10 mM H_2_O_2_, 100 M methyl jasmonate (MeJA), 500 μM ethephon (ETH) and 1 mM salicylic acid (SA). The mechanical damage treatment involved cutting the upper half of the needle, and the rest of the treatments were spray treatments. Three uniformly growing seedlings were selected for each treatment as three biological replicates. The needles were taken as samples every 0 h, 3 h, 6 h, 12 h, 24 h and 48 h, immediately frozen in liquid nitrogen and stored at −80 ℃. Samples collected at 0 h without any treatment were used as controls.

### 2.5. RNA-Seq Data Analysis of PmWRKY Genes

Information about CO_2_ treatment of *P. massoniana* for Illumina RNA-seq is provided below. In brief, one-year-old *P. massoniana* seedlings were used in this study. Individuals of the same clones with similar heights, uniform growth and strong growth potential were selected as the test materials and subsequently moved into a growth chamber. The growth conditions were 10 h light/14 h dark cycles at 25 °C in the chamber. Air containing approximately twice the CO_2_ concentration in the chamber before the experiment was aerated into the growth chamber constantly for at least 24 h. The CO_2_ concentration in the chamber was monitored by an infrared CO_2_ analyzer (SenseAir, Delsbo, Sweden). The seedlings were sampled after 6 h, 12 h and 24 h of treatment with the high CO_2_ concentration [15]. For each treatment, leaves collected from three triangular bottles were sampled as three biological replicates for RNA-seq. The mixed RNA sample (mixture combining treated plant samples from all time points) was sent for Iso-Seq. Fragments per kilobase of exon model per million reads mapped (FPKM) values were calculated to estimate the abundance of *P. massoniana* WRKY gene transcripts. The relative expression of control check(CK) was set as "1". Online OmicShare tools were used to create heatmaps based on the values of log_2_ (FPKM +0.01).

### 2.6. RNA Extraction and Quantitative Real-Time Reverse Transcription PCR 

Total RNA was extracted using the RNAprep Pure Kit (DP441, Tiangen Biotech, Beijing, China). RNA concentration and purity were measured with a NanoDrop 2000 (Thermo Fisher Scientific, Waltham, MA, USA), and RNA integrity was estimated by 1.2% agarose gel electrophoresis [22]. First-strand cDNA was synthesized using the One-step gDNA Removal and cDNA Synthesis Kit (AT311, TransGen Biotech, Beijing, China). Primers for quantitative real-time reverse transcription PCR (qRT-PCR) were designed using Primer 5.0 (Data S1). SYBR Green reagents were used to detect the target sequence. Each PCR mixture (10 µL) contained 1 µL of diluted cDNA (20× dilution), 5 µL of SYBR Green Real-time PCR Master Mix, 0.4 µL of each primer (10 µM), and 3.2 µL of ddH_2_O. The PCR program had six stages: (1) 95 °C for 60 s (preincubation); (2) 95 °C for 15 s, (3) 60 °C for 15 s and (4) 72 °C for 10 s, repeated 40 times (amplification); (5) 95 °C for 0.5 s; and (6) 60 °C for 1 min (melt). The PCR quality was estimated based on melting curves. TUB (tubulin beta) was used as the internal control [22]. Three independent biological replicates and three technical replicates for each biological replicate were examined. Quantification was achieved using comparative cycle threshold (Ct) values, and gene expression levels were calculated using the 2^-∆∆Ct^ method. The significance was determined by the *t*-test using SPSS statistical software (IBM, New York, NY, USA) (* *p* < 0.05, ** *p* < 0.01).

## 3. Results

### 3.1. Verification of WRKY Proteins in P. massoniana

**Figure 1:**
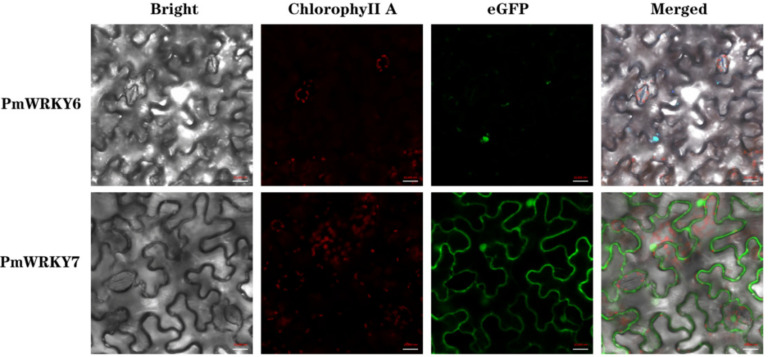
Subcellular localization of *PmWRKY6* and *PmWRKY7* in *Nicotiana benthamiana.*

Initially, 55 gene candidates corresponding to the WRKY family were obtained from the Pfam database. Further verification was performed using the CD-search program based on these gene models to ensure the presence of the WRKY domain. Following the removal of incorrectly predicted WRKY genes and redundant sequences, 31 genes were selected and annotated as *P. massoniana* WRKY genes. These WRKY genes were named *PmWRKY1*–*PmWRKY31* (Table 1). The coding sequences of validated *PmWRKY* genes are available in Supplementary Data S2. The number of amino acids in the predicted protein products varied from 99 to 788. Among the 31 *PmWRKY* proteins, *PmWRKY15*, with 330 amino acids, was the smallest, while the largest protein was *PmWRKY20* (788 amino acids). The range of protein molecular weights was 11.3–86.2 kDa, and the isoelectric point values ranged from 4.68 (*PmWRKY8*) to 9.99 (*PmWRKY26*). According to the results of CELLO and PSORT prediction of the subcellular localization of *PmWRKY1*-*PmWRKY31* proteins, almost all the proteins have a predicted nuclear localization. The prediction results show that 31 sequences have NLS or similar sequences. The prediction results for these proteins are shown in Table 1 and Table S2. In order to verify the nuclear localization, we transiently expressed *PmWRKY6* and *PmWRKY7* fused to GFP in *N. benthamiana* leaves and we analyzed their subcellular localization. The fluorescence signals of *PmWRKY6/7*:GFP were observed on the nucleus (Figure 1). 

### 3.2. Phylogenetic Analysis of PmWRKY Proteins

**Figure 2:**
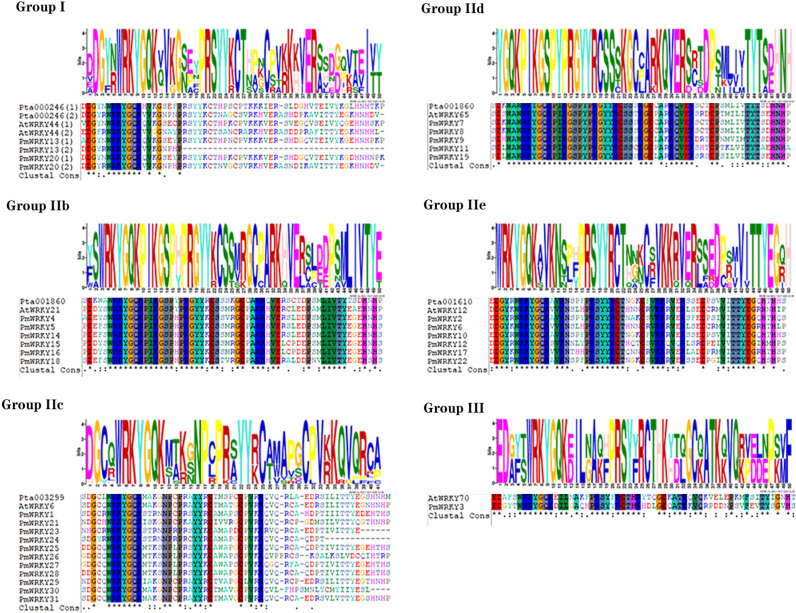
Multiple sequence alignment of *PmWRKY* domains.

**Figure 3:**
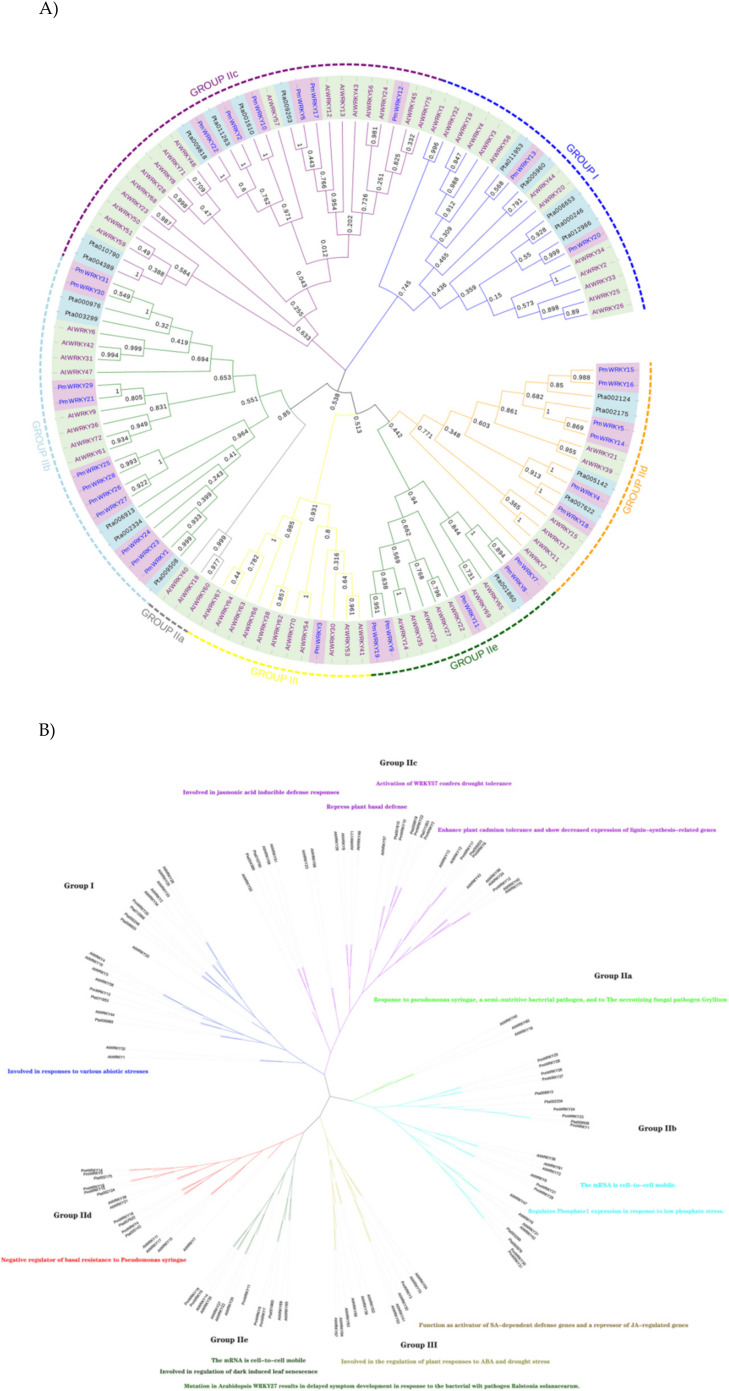
Classification of different groups of *PmWRKY*s, *AtWRKY*s and *PtWRKY*s. Different color branches and strips are used to distinguish different subgroups. In addition, the purple background is *P. massoniana*, the blue background is *P. taede* and the yellow background is *A. thaliana.* (**A**) The maximum likelihood method was used to analyze the evolutionary trees of 31 *PmWRKY*s, 65 *AtWRKY*s and 21 *PtWRKY*s. (**B**) The functions of each *AtWRKY* group marked in the evolutionary tree [24,25,26,27,28].

Based on the classification in *Arabidopsis*, 65 *AtWRKY* proteins from various groups or subgroups were randomly selected as representatives for comparison with *P. massoniana* and *P. taeda* based on previous studies [14,23]. The most prominent feature of WRKY proteins is the 60-amino-acid WRKY domain, which comprises the highly conserved signature "WRKYGQK" followed by a C2H2- or C2HC-type zinc finger motif [4]. As a result, the alignment of the *PmWRKY* proteins was examined based on their WRKY domains, which span ~60 amino acids. According to the combined alignment results, *PmWRKY* basically contains the conserved heptapeptide "WRKYGQK" in its domain sequence (Figure 2). The phylogenetic relationship among the WRKY domains of the *PmWRKY*, *PtWRKY* and *AtWRKY* proteins was studied as shown in Figure 3. The phylogenetic analysis indicated that *PmWRKY* proteins can be divided into three major groups, corresponding to groups I, II and III, when compared with the proteins from *A. thaliana* [3]. Of the 31 *PmWRKY* proteins, 2 belong to group I, 28 to group II, and 1 to group III. In group II, WRKY proteins can further be classified into five subgroups: 0 *PmWRKY* proteins belong to IIa, 11 to IIb, 6 to IIc, 6 to IId and 5 to IIe (Table 2).

### 3.3. Compositions of PmWRKY Protein Motifs

**Figure 4:**
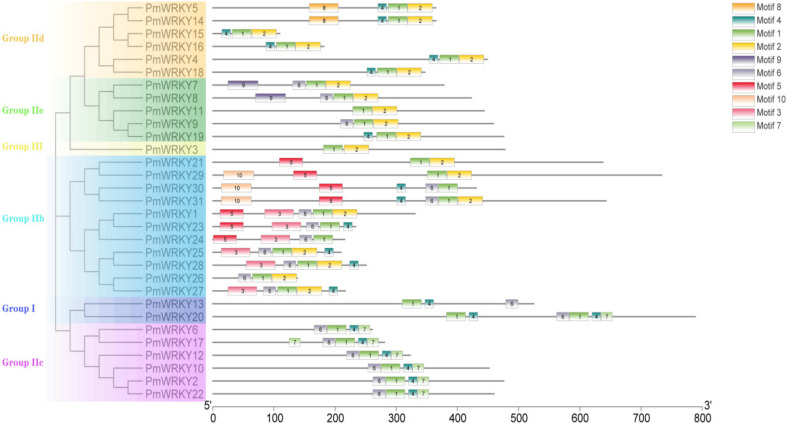
Visualization of classification of 31 *PmWRKY* proteins. The motifs present in the PmWRKY proteins based on MEME analysis. Architecture of 10 conserved protein motifs in *PmWRKY*s. Each motif is represented in a different color (Motif 1–10).

The conserved shared motifs were determined using the full-length open reading frames (ORFs) of *PmWRKY* proteins by the MEME program. Ten motifs were identified in the 31 *PmWRKY* protein sequences. The amino acid length of the 10 motifs ranged from 15 to 50. The pattern of the conserved motifs is listed in Table 3 and illustrated in Figure 4. Motif 1 and Motif 2 formed the main structure of the WRKY domain ("WRKYGQK"); motif 1 was identified in all *PmWRKY* proteins, while motifs 3, 5 and 10 were observed only in group IIb. As shown in Figure 4, in addition to the widely distributed WRKY domain motifs 1, 2 and 4, the same group of *PmWRKY* members usually had similar motif compositions (Table 3). Motif 8 was exclusively found in group IId, whereas motif 9 was observed only in group IIe. Motif 6 was observed in groups I, IIb, IIc, and IIe, and motif 7 was observed in groups I and IIb. The functions of most of these motifs remain to be elucidated. A similar motif arrangement for *PmWRKY* proteins in a subpopulation indicated that particular subfamilies had conserved structures. Coupled with the results from the phylogenetic tree, the conserved composition of *PmWRKY* proteins in the same groups or subgroups strongly supports the reliability of these group classifications.

### 3.4. Analysis of the Transcriptional Profiles of PmWRKY Genes

**Figure 5:**
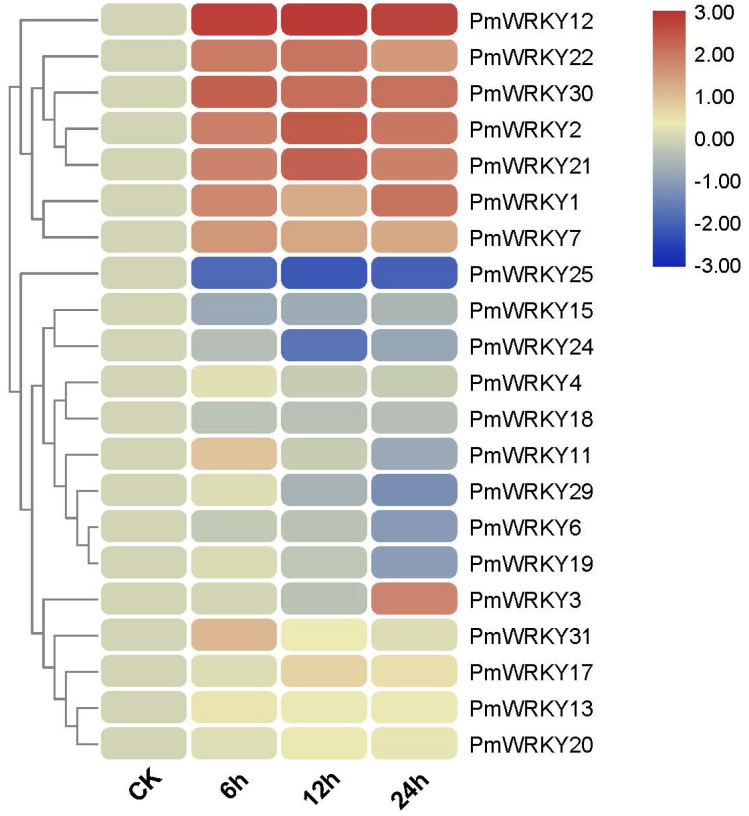
Transcriptional profiles of WRKY family members in *P. massoniana* under elevated CO_2_ stress, which includes four sets of data: 0 h(CK), 6 h, 12 h and 24 h after treatment. The relative expression of CK was set as "1". The color scale represents relative expression levels based on the values of log_2_ fold change scale.

The sequence and heat map data of this experiment were based on CO_2_ stress transcriptome (Figure 5). The expression levels of some genes in the transcription group under CO_2_ stress were too low to be detected, so only 21 expressions of *PmWRKY* were given. The expression of some genes exhibited significant trends during treatment with CO_2_ stress. For instance, the expression of *PmWRKY25* under CO_2_ stress was kept lower than control; whereas, the transcriptions of *PmWRKY1*, *PmWRKY2*, *PmWRKY7*, *PmWRKY12*, *PmWRKY21*, *PmWRKY22* and *PmWRKY30* were increased under CO_2_ stress treatment. In addition, the expression levels of some genes change significantly at some point in time. The expression level of *PmWRKY24* was also significantly lower than that of the control at 12h. The expression level of *PmWRKY3* was up-regulated at 24h. The results indicate that these genes may be regulated by CO_2_ stress. To obtain insights into the potential roles of *PmWRKY* genes, the expression levels of *PmWRKY*s were further determined by quantitative real-time PCR in leaves under treatments with three hormones (i.e., ETH, MeJA and SA), one abiotic stressor (i.e., H_2_O_2_) and mechanical damage stress. In order to analyze the transcription level of *PmWRKY* genes under different stresses, six *PmWRKY* genes with potential of resistance to stresses and high expression level in the transcriptome were selected for qRT-PCR through homologous clustering of *AtWRKY* genes related to stress resistance (Table S3).

**Figure 6:**
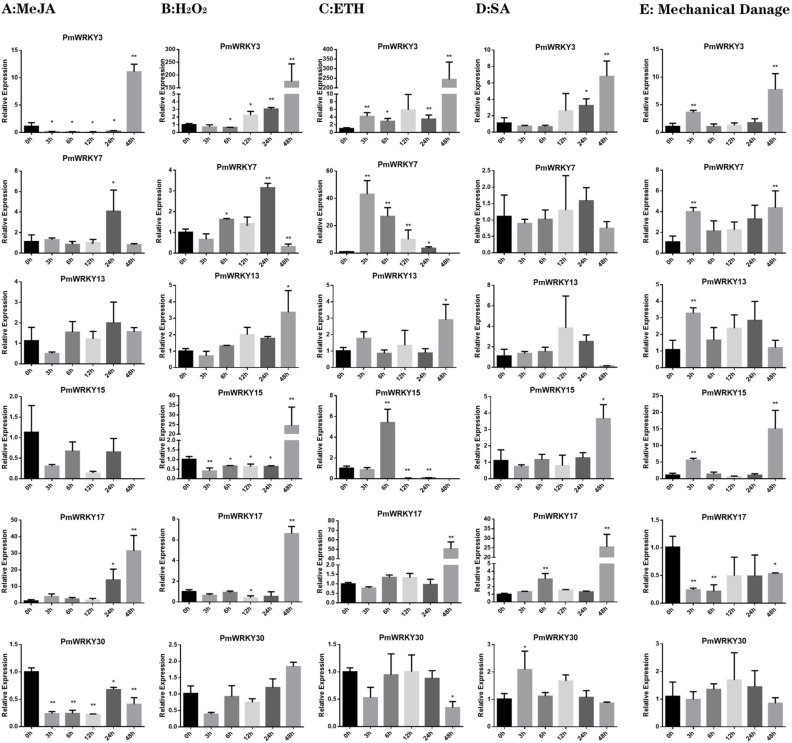
Differential transcription of *PmWRKY* genes in *P. massoniana* leaves under abiotic treatments and exogenous hormone supplies. (**A**) MeJA, (**B**) H_2_O_2_, (**C**) ETH, (**D**) SA, (**E**) Mechanical damage stress. The color scale represents relative expression levels based on the values of log_2_ (2^−∆∆CT^). Asterisks represent significant differences between each time point and 0 h (* *p* < 0.05, ** *p* < 0.01). MeJA, methyl jasmonate; H_2_O_2_, hydrogen peroxide; ETH, ethephon; SA, salicylic acid. Color images are available online.

Under MeJA treatment, the general trend of expression of *PmWRKY3*, *PmWRKY*7 and *PmWRKY17* were increasing. It is worth noting that the expression of *PmWRKY3* decreased at 3–24 h, and the expression level of *PmWRKY7* peaked at 24 h and then decreased. The expressions of *PmWRKY13* and *PmWRKY15* were relatively stable, and that of *PmWRKY30* was generally down-regulated. Among these genes, *PmWRKY3*, *PmWRKY*7, *PmWRKY17* and *PmWRKY30* were more sensitive to MeJA induction. In general, H_2_O_2_ treatment induced the expression of *PmWRKY*s(*PmWRKY3*, *PmWRKY7*, *PmWRKY13*, *PmWRKY15* and *PmWRKY17*) to be up-regulated and peak at 48 h (Except that *PmWRKY7* peaks at 24 h). In addition, the expression of *PmWRKY30* was relatively stable and had no significant tendency. Under ETH treatment, the expression trends of *PmWRKY*s were diverse (Figure 6C). The expressions of *PmWRKY3*, *PmWRKY13* and *PmWRKY17* were up-regulated after treatment. However, the expressions of *PmWRKY13* and *PmWRKY17* were relatively stable during 3–24 h, while *PmWRKY3* was up-regulated steadily from 3 h. The expression of *PmWRKY7* and *PmWRKY15* first increased to the highest level and then decreased. The expression of *PmWRKY30* was stable in the early stage and decreased at 48 h. Under SA treatment, the expression levels of *PmWRKY3*, *PmWRKY15* and *PmWRKY17* showed an overall increasing trend, while the expression level of *PmWRKY7* and *PmWRKY13* showed a stable trend. However, that of *PmWRKY30* increased to its maximum at 3 h and then restored stability. Under mechanical damage stress, the expression levels of the *PmWRKY3*, *PmWRKY7*, *PmWRKY13* and *PmWRKY15* increased at 3 h and then recovered to be stable, but the expression levels of *PmWRKY3*, *PmWRKY7* and *PmWRKY15* increased again and reached their maxima at 48 h. The expression of *PmWRKY17* was always lower than that of control after treatment, while the expression of *PmWRKY30* showed no obvious trend of change.

## 4. Discussion

Since the genome of *P. massoniana* is 200 times larger than that of *A. thaliana*, it is difficult to obtain the sequence in a short time, so we used the transcriptome to identify the WRKY gene family here. *PmWRKY* genes were searched for based on the Illumina RNA-seq and Iso-Seq databases in this study. A total of 31 genes, which were designated *PmWRKY1* to *PmWRKY31*, were identified. With the aim of improving *P. massoniana*, this systematic analysis lays the foundation for further research into the functions of WRKY genes.

In this study, the same group of *PmWRKY* proteins in the phylogenetic tree shared common motifs based on the MEME analysis results with default parameters, indicating that the proteins were highly conserved and strongly supporting the reliability of the group classifications [29,30]. The number of WRKY gene family members in plants increased rapidly as the systems evolved from lower single-celled eukaryotic algae to higher multicellular angiosperm plants. Group I may be the ancestor, transitioning to group II by losing or splitting the structural domain. Most of the studied WRKY TFs belong to group II [3]. Group III may have been produced from group II through substitution of the H (histidine) of the zinc finger structure to a C residue. As a result, groups I and II are more highly conserved [31]. Among the 31 *PmWRKY* proteins identified, only one domain belonged to group III. The possible reasons are that the databases are not complete and that *PmWRKY* protein sequences belonging to group III were incomplete and manually discarded. Another reason for the lack of group III proteins may be that the screening criteria were too stringent and the E value cut-off (set to 10^−3^) was too strict. Previous studies have reported that the woody plants evolved a lower number of WRKY genes than herbaceous plants [32], and the evolutionary loss of the WRKY domain in dicotyledons was less than that in monocotyledons [33]. We can speculate further that *P. massoniana*, which is a gymnosperm, underwent a high degree of evolutionary loss of the WRKY domain, similar to *P. taeda* (Table 3). As shown in Figure 1, the *PmWRKY* proteins from groups IIa and IIb present a close relationship, while groups IId and IIe are closer together. Since the split of IIa and IIb and the split of IId and IIe occurred much later than those of other groups in the ancestor of land plants [3], IIa and IIb should likely be merged into a single subfamily that incorporates subgroups IId and IIe [34]. With evolution from lower plants to higher plants and from the aquatic environment to the terrestrial environment, plants have established signal transduction pathways related to growth, development, morphogenesis, metabolic regulation and stress resistance.

In order to verify the nuclear localization, we randomly selected two genes for subcellular localization, *PmWRKY6* and *PmWRKY7*, which have localization on the nucleus, whereas *PmWRKY7* also seems to be localized in other locations that are compatible with the cytoplasm (Figure 1). We speculated that *N. benthamiana* has a huge vacuole that sometimes displaces/pushes the cytoplasmic proteins against the membrane, which means that GFP-cytoplasmic proteins could be observed as "false membrane localized proteins." In addition, the possible reason is that the function or localization of transcription factors may be affected by other transcription factors. For example, *GL1* is located in the nucleus. However, in the co-localization study, the interaction between *AtMYC* and *GL1* leads to *GL1* localization to the cytoplasm [35]. 

Since domains and motifs are related to transcriptional activity and protein interactions, generally, the function and characteristics of TFs can be determined by domain and motif analyses [36]. The results of conservative domain analysis showed that motifs 1 and 2 are related to a complete WRKY domain including a WRKY heptapeptide domain and a zinc finger structure and motifs 1 exist in all WRKY subfamily members. This sequence is essential for WRKY transcription factor recognition and binding to the W-box element at the target gene promoter [37]. Previous studies have reported variations in WRKYGQK sequences in different plants, which may lead to WRKY transcription factor recognition and binding to cis-regulatory elements other than W-Box [38]. There are three types of mutations in the WRKY heptapeptide domain. The first type is the second site variation of heptapeptide domain, usually from Rrg(R) to Lys(K). Namely, WRKYGQK mutates to WKKYGQK. The second type is the sixth site from Glu(Q) mutation to Glu(E) or Lys(K), namely WRKYGQK mutation to WRKYGEK or WRKYGKK. The third type shows the overall loss of C-terminal WRKY heptapeptide domain. According to the study, the second type of mutation is the common mutation type of WRKY gene family, which has been reported in many species, such as *Prunus persica* [39], *Populus* [40] and *Cunninghamia lanceolata* [41]. There is ample evidence that the WRKY gene family is important in the regulation of plant growth and development [42,43,44,45]. In addition, some WRKY proteins act as negative regulators of plant defense by binding to the DNA sequence TGAC [46]. Since gene expression patterns can provide important clues regarding gene function, we used qPCR to detect the expression of *PmWRKY* genes in the leaves of 2-year-old *P. massoniana* seedlings. The expression profiles showed variations in *PmWRKY* expression in different treatments. Our study showed that six *PmWRKY*s (*PmWRKY3*, *PmWRKY7*, *PmWRKY13*, *PmWRKY15*, *PmWRKY17* and *PmWRKY30*) responded to at least two types of stress, among which *PmWRKY3* was significantly elevated by induction with MeJA, SA, ETH, H_2_O_2_, and mechanical stress. Interestingly, the expression of *PmWRKY3* peaked at 48 h under all the treatments. This suggests that the expression of these genes may be “time-specific”. In addition, some pairs of WRKY homologues have evolved different functionalities among different species [6], so little can be concluded by comparing WRKY genes with homologues. For example, the expression of *WRKY42* was significantly reduced under 10 mM H_2_O_2_ and 500 μM SA treatments at 1, 3 and 12 h, whereas *PmWRKY30* was not inhibited under MeJA and H_2_O_2_ treatment. Therefore, it is necessary to verify the function of genes through experiments in the later stage. In this study, we obtained four candidate genes (*PmWRKY3*, *PmWRKY7*, *PmWRKY15*, *PmWRKY17*) that are highly expressed in multiple stressors and deserve further exploration. The regulatory mechanism of WRKY genes under hormone, abiotic and mechanical stressors is complex, and the study of *PmWRKY* gene expression profiles will further promote functional research on the stress responses and signaling pathways of conifer trees in the future.

## 5. Conclusions

Comprehensive analysis of the *PmWRKY* genes was carried out in *P. massoniana*. Thirty-one *PmWRKY* genes were identified and divided into three major groups based on the presence of certain amino acid motifs. *PmWRKY* genes are important for the growth and development of *P. massoniana*, as shown by their expression levels under various treatments. *PmWRKY* gene expression is not completely consistent with the expression pattern of the corresponding genes in *A. thaliana*. This phylogenetic and gene expression analysis sheds light on the functions of *PmWRKY* genes in this specific conifer tree. This study will provide a theoretical basis for functional studies of the *WRKY* gene family, be very useful for understanding the biological role of individual WRKY gene in *P. massoniana* and provide a potential strategy for further breeding *P. massoniana*.

## Figures and Tables

**Table 1:** Summary of *PmWRKY* Sequences.

Gene ID	Gene	cDNA Length	Aa	MW(kDa)	pl	Group	WRKY Domain	Subcellular Localization	NLS
m.15387	*PmWRKY1*	996	331	37.6	8.9	IIb	WRKYGQK	Nucleus	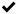
m.7589	*PmWRKY2*	1428	475	50.9	5.82	IIc	WRKYGQK	Nucleus	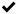
m.7738	*PmWRKY3*	1434	477	52.5	5.56	III	WRKYGQK	Nucleus	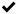
m.6876	*PmWRKY4*	1347	448	48.8	9.41	IId	WRKYGQK	Nucleus	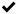
m.8763	*PmWRKY5*	1092	364	40.4	9.68	IId	WRKYGQK	Nucleus	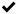
m.256914	*PmWRKY6*	783	261	29.8	6.49	IIc	WRKYGQK	Nucleus	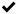
m.293152	*PmWRKY7*	1134	377	41.0	4.75	IIe	WRKYGQK	Nucleus	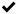
m.293159	*PmWRKY8*	1269	422	45.7	4.68	IIe	WRKYGQK	Nucleus	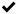
m.199902	*PmWRKY9*	1377	458	50.1	6.22	IIe	WRKYGQK	Nucleus	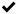
m.140205	*PmWRKY10*	1359	452	50.5	6.43	IIc	WRKYGQK	Nucleus	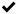
m.257305	*PmWRKY11*	1332	443	48.0	6.47	IIe	WRKYGQK	Nucleus	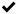
m.115513	*PmWRKY12*	969	322	36.4	9.18	IIc	WRKYGQK	Nucleus	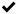
m.62912	*PmWRKY13*	1578	525	55.7	8.95	I	WRKYGQK X2	Nucleus	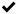
m.156607	*PmWRKY14*	1095	364	40.4	9.68	IId	WRKYGQK	Nucleus	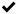
m.423632	*PmWRKY15*	330	233	26.2	9.89	IId	WRKYGQK	Nucleus Cytoplasmic	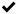
m.423648	*PmWRKY16*	546	181	20.1	9.55	IId	WRKYGQK	Nucleus	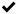
m.59196	*PmWRKY17*	843	280	32.3	6.83	IIc	WRKYGQK	Nucleus	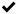
m.416602	*PmWRKY18*	1041	346	37.4	9.42	IId	WRKYGQK	Nucleus	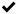
m.318109	*PmWRKY19*	1425	475	50.8	5.37	IIe	WRKYGQK	Nucleus	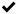
m.198227	*PmWRKY20*	2367	788	86.2	9.02	I	WRKYGQK X2	Nucleus	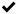
m.175245	*PmWRKY21*	1914	637	70.1	6.77	IIb	WRKYGQK	Nucleus	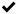
m.394091	*PmWRKY22*	1380	459	50.2	6.28	IIc	WRKYGQK	Nucleus	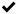
m.282372	*PmWRKY23*	705	234	27.1	8.62	IIb	WRKYGQK	Nucleus	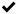
m.282362	*PmWRKY24*	651	216	24.9	9.66	IIb	WRKYGQK	Nucleus	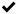
m.50488	*PmWRKY25*	633	210	23.8	9.47	IIb	WRKYGQK	Nucleus	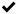
m.50492	*PmWRKY26*	417	138	15.7	9.99	IIb	WRKYGQK	Nucleus	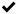
m.50499	*PmWRKY27*	654	217	24.4	9.27	IIb	WRKYGQK	Nucleus	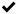
m.50472	*PmWRKY28*	756	251	28.6	9.43	IIb	WRKYGQK	Nucleus	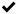
m.57136	*PmWRKY29*	2202	733	79.5	6.39	IIb	WRKYGQK	Nucleus	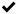
m.252813	*PmWRKY30*	1293	430	47.5	5.74	IIb	WRKYGQK	Nucleus	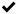
m.252861	*PmWRKY31*	1929	642	69.4	6.06	IIb	WRKYGQK	Nucleus	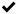

**Table 2:** Comparison of groups between *P. massoniana* and *P. taeda.*

Group	Subgroup	Gene Number	
		PmWRKY	PtWRKY
I		2	5
II	IIa	0	0
	IIb	11	5
	IIc	6	6
	IId	6	4
	IIe	5	1
III		1	0
Total		31	21

**Table 3:** Details of conserved motifs from *PmWRKY* proteins.

Motif	Width	Motif Sequence
1	32	SEADIPSDGYRWRKYGQKPVKGSPYPRSYYRC
2	41	SSARGCPARKQVERCATDPSILITTYEGEHNHSWPLSANAS
3	48	WDCLEQGWEKDNKNAKFMDDQQLPSSKRTLNYFQSAQIENRINSSTDD
4	15	RVKKRVERTIDDPAI
5	39	QVEINRMKEENQNLKSMLSRMINNYHNLQMHMMSVMQQQ
6	21	KKHKVKGRRTIRVPRFIVSTR
7	19	VITTYEGQHTHPSPALLRS
8	48	ADTNRHQQLHPQMHYPPLQLQHLSPQPEVMFRNGYMQLDNSMSCTATI
9	50	RCAATCLGGVAALYPEKQENSCNQRNEGEFMFGTSIVKQELEDQLDFVQP
10	50	VRELLDTELKQKCRRKGDFMADAPRVDRLGGIDLSVKLEETENEEKLMTD

## Supplementary Materials

The following are available online at https://www.mdpi.com/2073-4425/11/11/1386/s1, Table S1: Primer pairs for quantitative polymerase chain reaction, Table S2: Subcellular localization prediction of *PmWRKY* proteins, Table S3: Homologous genes in *Arabidopsis thaliana*.
