# Supplementary material for: Transcriptome-Wide Identification of WRKY Transcription Factors and Their Expression Profiles under Different Types of Biological and Abiotic Stress in Pinus massoniana Lamb
Source: Genes (Basel). 2020 Nov 23;11(11):1386. doi: 10.3390/genes11111386 (PMC7700256; doi:10.3390/genes11111386)
Supplement: Supplementary file 1 [file genes-11-01386-s001.pdf]

genes

MDPI

Article

Transcriptome-Wide Identification of WRKY Transcription Factors and Their Expression Profiles under Different Types of Biological and Abiotic Stress in *Pinus massoniana* Lamb

Sheng Yao [1], Fan Wu [1], Qingqing Hao [1] and Kongshu Ji [1,*]

Supplementary Materials

Table S1. Primer pairs for quantitative polymerase chain reaction.

Gene ID	Primer5'→3'
PmWRKY3	F：TACAAGGCAGTGAGAAAGAG
	R：AGGTGGAGGCAGGAAATTAG
PmWRKY7	F：TCTGATAATCTCTTCTCGCT
	R：GTTCCTGTTCTCGTCGTCCT
PmWRKY13	F：CACAATCATCCCAAACCTCA
	R：GAACCATCCACATTCCTTCC
PmWRKY15	F：ACAAAAAGGTGGGAGTAAGG
	R：AAATGTGCTAGTAGCGGAGG
PmWRKY17	F：CACAGACACAATATCCCACA
	R：TTCACATCACAACTAGACGA
PmWRKY30	F：TGCTCTGCCCTCATTTCTCCC
	R：AAATCGCCTTTTCTTCTGCACT

Table S2. Subcellular localization prediction of *PmWRKY* proteins.

SeqID	PSORT	CELLO (reliable-index > 1)	Same result
PmWRKY1	nucl: 14	Nuclear	Nuclear
PmWRKY2	nucl: 14	Nuclear	Nuclear
PmWRKY3	nucl: 13, vacu: 1	Nuclear	Nuclear

PmWRKY4	nucl: 14	Nuclear	Nuclear
PmWRKY5	nucl: 10.5, cyto_nucl: 6, mito: 1, plas: 1, cysk: 1	Nuclear	Nuclear
PmWRKY6	nucl: 11, chlo: 1, cyto: 1, plas: 1	Nuclear	Nuclear
PmWRKY7	nucl: 12.5, cyto_nucl: 7.5, cyto: 1.5	Nuclear	Nuclear
PmWRKY8	nucl: 13, cyto: 1	Nuclear	Nuclear
PmWRKY9	nucl: 13, chlo: 1	Nuclear	Nuclear
PmWRKY10	nucl: 14	Nuclear	Nuclear
PmWRKY11	nucl: 8, chlo: 4, cyto: 1, mito: 1	Nuclear	Nuclear
PmWRKY12	nucl: 12.5, cyto_nucl: 7, chlo: 1	Nuclear	Nuclear
PmWRKY13	nucl: 14	Nuclear	Nuclear
PmWRKY14	nucl: 10.5, cyto_nucl: 6, mito: 1, plas: 1, cysk: 1	Nuclear	Nuclear
PmWRKY15	cyto: 7, nucl: 3, mito: 2, chlo: 1, extr: 1	Nuclear	Nuclear Cytoplasmic
PmWRKY16	nucl: 7, chlo: 3, mito: 3, extr: 1	Nuclear	Nuclear
PmWRKY17	nucl: 11, chlo: 1, extr: 1, vacu: 1	Nuclear	Nuclear
PmWRKY18	nucl: 14	Nuclear	Nuclear
PmWRKY19	nucl: 12, cyto: 1, cysk: 1	Nuclear	Nuclear
PmWRKY20	chlo: 9, nucl: 5	Nuclear	Nuclear
PmWRKY21	nucl: 14	Nuclear	Nuclear
PmWRKY22	nucl: 14	Nuclear	Nuclear
PmWRKY23	nucl: 10.5, cyto_nucl: 6.5, chlo: 2, cyto: 1.5	Nuclear	Nuclear
PmWRKY24	nucl: 14	Nuclear	Nuclear

PmWRKY25	nucl: 14	Nuclear	Nuclear
PmWRKY26	nucl: 12, chlo: 1, plas: 1	Nuclear	Nuclear
PmWRKY27	nucl: 13, chlo: 1	Nuclear	Nuclear
PmWRKY28	nucl: 12.5, cyto_nucl: 7.5, cyto: 1.5	Nuclear	Nuclear
PmWRKY29	nucl: 13, cyto: 1	Nuclear	Nuclear
PmWRKY30	nucl: 10, cyto: 3, chlo: 1	Nuclear	Nuclear
PmWRKY31	nucl: 13, cyto: 1	Nuclear	Nuclear

Table S3. Homologous genes in *Arabidopsis thaliana.*

Gene	Orthologs	Treatment	Reference
PmWRKY3	*AtWRKY30*	heat stress, drought, jasmonic acid	[1,2]
PmWRKY7	*AtWRKY55*	SA	[3]
PmWRKY13	*AtWRKY44*	drought	[4]
PmWRKY15	*AtWRKY21*、 *AtWRKY39*	osmotic stress, heat stress	[5,6]
PmWRKY17	*AtWRKY13*	cadmium tolerance	[7]
PmWRKY30	*AtWRKY6*, *AtWRKY42*, *AtWRKY47*	ABA, SA, selenium H_2O_2, tolerance	[8,9,10]

References

1. El-Esawi, M.A.; Al-Ghamdi, A.A.; Ali, H.M. Overexpression of *AtWRKY30* Transcription Factor Enhances Heat and Drought Stress Tolerance in Wheat (*Triticum aestivum L.*). *Genes* **2019**, *10*, 163.
2. Peng, X.X.; Hu, Y.J.; Tang, X.K. Constitutive expression of rice *WRKY30* gene increases the endogenous jasmonic acid accumulation, PR gene expression and resistance to fungal pathogens in rice. *Planta* **2012**, *236*, 1485–1498.
3. Wang, Y.Q.; Cui, X.; Yang, B. *WRKY55* transcription factor positively regulates leaf senescence and defense response through modulating the transcription of genes implicated in ROS and SA biosynthesis in *Arabidopsis*. *Development (Cambridge, England)* **2020**, *147*, doi: 10.1242/dev.189647.
4. Han, Y.Y.; Zhang, X.; Wang, W. The suppression of *WRKY44* by *GIGANTEA*-miR172 pathway is involved in drought response of *Arabidopsis thaliana*. *PLoS ONE* **2017**, *8*, e73541.
5. Zhao, K.X.; Chu, S.S.; Zhang, X.D. *AtWRKY21* negatively regulates tolerance to osmotic stress in *Arabidopsis*. *Environ. Exp. Bot.* **2020**, *169*, 103920.
6. Li, S.J.; Zhou, X.; Chen, L.G. Functional characterization of *Arabidopsis thaliana WRKY39* in heat stress. *Mol. Cells* **2010**, *29*, 475–483.
7. Sheng, Y.B.; Yan, X.X.; Huang, Y. The WRKY transcription factor, *WRKY13*, activates PDR8 expression to positively regulate cadmium tolerance in *Arabidopsis*. *Plant Cell Environ.* **2019**, *42*, 891–903.

8. Huang, Y.; Feng, C.Z.; Ye, Q. *Arabidopsis WRKY6* Transcription Factor Acts as a Positive Regulator of Abscisic Acid Signaling during Seed Germination and Early Seedling Development. *PLoS Genet.* **2016**, *12*, e1005833.
9. Niu, F.; Cui, X.; Zhao, P. *WRKY42* transcription factor positively regulates leaf senescence through modulating SA and ROS synthesis in *Arabidopsis thaliana*. *Plant J.* **2020**, *104*, 171–184.
10. Wu, X.; Tao, M.; Meng, Y. The role of *WRKY47* gene in regulating selenium tolerance in *Arabidopsis thaliana*. *Plant Biotechnol. Rep.* **2020**, *14*, 121–129.

Publisher's Note: MDPI stays neutral with regard to jurisdictional claims in published maps and institutional affiliations.

© 2020 by the authors. Licensee MDPI, Basel, Switzerland. This article is an open access article distributed under the terms and conditions of the Creative Commons Attribution (CC BY) license (http://creativecommons.org/licenses/by/4.0/).
